# What mRNA Abundances Can Tell us about Metabolism 

**DOI:** 10.3390/metabo2030614

**Published:** 2012-09-12

**Authors:** Andreas Hoppe

**Affiliations:** Institute for Biochemistry, Charité University Medicine Berlin, Charitéplatz 1, Berlin 10117, Germany; Email: hoppe@bioinformatics.org; Tel.: +49-30-450-528176

**Keywords:** transcriptomics, metabolic network, metabolism, metabolic function, Virtual-Liver Network

## Abstract

Inferring decreased or increased metabolic functions from transcript proﬁles is at ﬁrst sight a bold and speculative attempt because of the functional layers in between: proteins, enzymatic activities, and reaction ﬂuxes. However, the growing interest in this ﬁeld can easily be explained by two facts: the high quality of genome-scale metabolic network reconstructions and the highly developed technology to obtain genome-covering RNA proﬁles. Here, an overview of important algorithmic approaches is given by means of criteria by which published procedures can be classiﬁed. The frontiers of the methods are sketched and critical voices are being heard. Finally, an outlook for the prospects of the ﬁeld is given.

## 1. Introduction

Genetic regulation is a major control mechanism of the activity of the cell’s metabolic functions, especially in the frame of longer times where the metabolic function is described as a speciﬁc metabolic input/output behavior of the cell. Its activity is deﬁned as the metabolic ﬂux, *i.e*., the consumption and production rate of speciﬁc metabolites, related to this function. A general description of the ﬂow of information from the genome to metabolism is as follows: the process is initiated by transcription factors, RNA polymerase transcribes genes into RNA, RNA is transported to the ribosome and translated into proteins, and after folding, post-transcriptional modiﬁcation, and transport to the site of action, proteins act as enzymes and transporters catalyzing biochemical reactions ﬂuxes of the molecules in the cell (quantiﬁed by the reaction *ﬂux*, the net number of converted molecules by time by cell volume). The mechanisms of this control have already been recognized as being complex and multi-level—setting up predictive quantitative models is difﬁcult [1]. Transcription factors are controlled by mechanisms on different layers of the cellular system: their own transcription, translation, and post-translational modiﬁcation, their localization, their activation by external signaling substances or the concentration of internal metabolites, and their combination with other transcription factors. Some mRNA species [2] and transcription factors [3] are directionally transported by microtubuli in a controlled manner while others rely on diffusion. The efﬁciency of the translation may dramatically differ between different genes [4]. Finally, the catalytic efﬁciency of different enzymes varies along six orders of magnitude [5]. The metabolic ﬂux rate is not only determined by the enzymes’ concentrations but by a multitude of regulators, some of which change the reaction rate by several orders of magnitude [5]. For many processes the modifying factors have been discovered but on the scale of the whole genome, most of them are unknown. 

It might seem presumptuous to propose that transcript data can be used to predict metabolic functions. However, these predictions have received much interest because:

- the layer of RNA transcripts (as opposed to the layer of proteins, the layer of reaction ﬂuxes, and the layer of metabolites) is the only layer where a complete quantitative snapshot of all molecular species is currently feasible. Reaction ﬂux estimations currently cover only a tiny share of all reactions. Metabolite concentrations are currently measured for some 100 s metabolites but speciﬁc classes of metabolites such as lipids still present large challenges. Protein amount estimations at the genome-scale are now being done but the effort necessary is huge. The layer of DNA, whose information is a precondition for any transcription-based analysis, is not mentioned as qualitative information.
- transcript arrays are moderately priced in relation to the amount of data gathered,
- the experimental effort for the researcher is moderate due to an highly automatized process,
- the technology provides a low ambiguity and accurate estimates of the RNA amount changes [6]. The high number of probes allows to distinguish between the RNA of separate genes, with only few exceptions. Ambiguity of the peaks is the main problem of the estimation of metabolite concentrations by mass spectrometry. Ambiguity is also the largest challenge in ﬂux estimations based on ^13^C marked substrates, and
- well-curated genome-scale reconstructions of the metabolic networks are available [7,8,9,10].

The measurement of reaction ﬂuxes, metabolite concentrations, enzyme activities, and protein amounts are currently undertaken for a subset of all molecular species. The measurement of protein amounts is just becoming feasible with the advent of techniques such as single-shot ultra HPLC [11]. *If* all enzyme activities and metabolite concentrations *were* available, a much more accurate prediction would be possible, but that is not the case on the large scale. Thus, to judge the results of the reviewed studies squarely it must be stressed that the *expectations must be lowered* accordingly. The systems biologist faces the trade-off between coverage versus accuracy versus the data being *closer* to the enzymatic activity, *i.e*., that quantitative proteomics data would provide a better indication of enzymatic activity, but the technique does not have the coverage provided by transcriptome data.

Here, studies with the primary focus on metabolism are reviewed. Other major areas of application of transcript data [12] are not covered, such as (i) detection of transcriptional co-regulation leading to (ii) detection of transcription factor binding sites [13] and (iii) transcriptional biomarkers [14]. 

## 2. Fundamental Studies

To demonstrate the difﬁculty of the task bridging several layers of cellular interaction, selected studies of the relation from one layer to the next will be sketched. 

### 2.1. Gene Chip Intensities→mRNA

DNA microarray read-outs depend on the RNA concentrations but also on the varying afﬁnity of the RNA to the probes, which is unknown on the large scale, thus special care is needed when analyzing the data [15]. Nevertheless, it is a very dependable technique [6]. For a comparison of different gene chip techniques, see Baldwin *et al*. [16]. Often, a genome-scale gene chip analysis is coupled with a more accurate qPCR for selected genes as a means of validation [17]. Advanced experimental techniques such as RNA-seq [18] and SAGE [19] allow a more accurate genome-scale quantiﬁcation of RNA than gene array readouts and will eventually replace them [20], but effort and price currently restrict its widespread use [21]. 

### 2.2. mRNA→Protein

In a pioneering study by Gygi *et al*. [22], the correlation between 106 studied mRNA levels to their coded protein levels showed a high value of 0.935. Gygi noted that the number is far lower if the extremely highly abundant proteins are disregarded; then it can be as low as 0.1. In a further note, the relation of protein levels below the detection limit to their respective mRNA levels is obviously unknown, thus, for the numerous proteins that only occur in very small quantities, the relation to RNA levels is unknown. In a subsequent study Grifﬁn *et al*. advocates the combined consideration of both mRNA and protein levels to understand the regulation of central metabolic functions in yeast [23,24]. Tuller *et al*. predicted protein abundances from mRNA expression levels by taking into account additional information on the genes [25]. The results on the test set showed a good correlation of 0.76. Further studies on the relation of RNA levels and protein abundances have been reviewed by Meier *et al*. [26]. In particular the study in human cell lines [1] should be mentioned. In an experimental analysis of Arabidopsis, among 319 protein/transcript pairs, 56% showed concurrence between transcript and protein, and it was suggested that for the others post-transcriptional modiﬁcation takes place [27]. 

Mechanistically, the relation between RNA and protein concentrations can be seen as the interplay of three aspects: (i) the life span of RNA and (ii) proteins as well as (iii) the translation efﬁciency at the ribosome [4]. In a groundbreaking work, Schwanhäusser studied the life cycles of RNA and protein translation in mammalian ﬁbroblasts and found “that the cellular abundance of proteins is predominantly controlled at the level of translation” [28]. The rates of mRNA synthesis and decay in yeast in response to stress have been measured [29] The life span of proteins *in vivo* has been assessed on a large scale in yeast [30] and for selected glycolytic enzymes in mammalian cells [31]. 

### 2.3. Enzyme Concentration → Enzyme Activity

The enzyme activity (the maximal catalytic rate vmax for a given cell volume) depends on the enzyme concentration. Mostly, the relation is approximately linear in a predeﬁned environment—the ratio is called turnover number. The turnover numbers of enzymes (together with other kinetic parameters) have been estimated for many enzymes, comprehensively reviewed and made available in public databases [5,32]. With respect to the set of all enzymes, this information is far from complete. Turnover numbers have been measured for different conditions (pH, temperature, and the concentrations of activators and inhibitors) and the resulting values vary considerably for one enzyme. Some enzymes are nine orders of magnitude more efﬁcient than others (minimal *vs.* maximal turnover numbers in [5]). Considering this data, the variability of this step in the chain from RNA to metabolic ﬂux is greater than of any of the other steps. 

### 2.4. Enzyme Activity → Metabolic Flux

The prediction of metabolic ﬂuxes from enzyme activity information (and concentration of reactants, products, and other metabolic species) has been extensively studied in the ﬁeld of kinetic modeling and its results are available in public databases [33,34]. A main challenge in the understanding is the interplay of metabolite concentration, enzyme levels, and reaction ﬂuxes in a highly connected network. The network effect, deﬁned as the difference of the simultaneous ﬂow of chemical reactions compared with the isolated ﬂow of reactions, modiﬁes the activity–ﬂux relation. It is studied in metabolic control analysis [35,36,37]. In extreme cases, it can lead to paradoxical situations where an increased enzyme amount leads to a lower ﬂux in the same metabolic reaction. 

### 2.5. Crossing Several Layers

Hancock *et al*. analyzed the relation of RNA abundance to metabolite concentrations in combination with the topological structure of the network. Based on clustering of correlated genes, their approach allows the identiﬁcation of hub reactions depending on a speciﬁc change of condition, which subsequently leads to a minimal set of commonly controlled metabolites. Their results support the hypothesis that the gene expression response (on different forms of stress on *E. coli* in this case) targets a small number of metabolites which consequently entails a large-scale change in the metabolism [38]. 

Kharchenko *et al*. found that the highest co-expression of metabolic genes is arranged in simple motifs in the metabolic network, in other words, “regulation of metabolic genes is local” [39]. Cakir *et al*. studied the transcriptional adaption of yeast on growth media. They calculate optimal transcript ratios on the basis of elementary ﬂux modes [40] and the comparison to real transcript ratios showed a high agreement [41]. This result, in comparison with other studies showing less agreement, leads to the conclusion that the adaptation on cellular substrates is a distinguished case. 

Hajduch *et al*. compared the proteome of different oilseed to reveal differences in the intermediary metabolism, and their analysis showed a diverging use of malate as a precursor for lipids [42]. Saito *et al*. reviewed studies using transcript and metabolite co-occurrence for various applications in plant biology [43]. Ishihama *et al*. performed a large-scale proteomic screening of *E. coli* and found that, among the enzymes, only proteins involved in energy metabolism are highly abundant [44]. 

Of particular interest are studies which measured RNA, protein, ﬂuxes, and metabolite concentrations in parallel in the same experiment [45,46]. The common ﬁnding in these studies is that there is not a high overall correlation between the abundance of RNA and the coded protein, between the enzyme and the catalyzed ﬂux, and between the metabolite concentrations and the level of enzymes that catalyze them. However, looking at the regulation of selected metabolic paths and functions, in almost all cases the pattern of abundance changes of RNA and protein is in accordance with the observed changes in reaction ﬂuxes and metabolite concentrations. To sum it up, although there is little direct predictivity of RNA to the reaction ﬂuxes, the transcriptional regulation of the metabolic function can still be observed in the RNA abundance data. 

### 2.6. mRNA → Fluxes

As a summary of an early attempt to relate transcript values to metabolic ﬂuxes, ter Kuile expressed “strong doubts on whether transcriptome and proteome analysis sufﬁces to assess biological function” [47]. The conclusion has been drawn by the authors of subsequent approaches that transcript proﬁles must be used in conjunction with other information to yield meaningful results. 

Moxley *et al*. [48] correlated the ﬂuxes (estimated by tracer experiments) to the respective RNA levels and found a mere correlation of 0.07, which could be increased to 0.8 by the use of a network-based model from which a parameter called “metabolite interaction density” is calculated. This density is used as a modiﬁer for the ﬂux prediction from RNA levels. The conclusion of this study is that the consideration of the metabolic network is *essential* to draw a predictive relation from transcript abundances to ﬂuxes. 

Yang *et al*. studied gene expression in Synechocystis in combination with ^13^C isotope-based ﬂux measurements and emphasizes the importance of integrating transcript and ﬂux data for the understanding of regulatory mechanisms [49]. 

Daran-Lapujade *et al*. studied the role of “hierarchical” ﬂux regulation (by changed enzyme activity, e.g., transcriptional regulation) versus metabolic regulation (change of ﬂux due to changed metabolite concentrations) for glycolytic enzymes in yeast [50]. Factor analysis showed that transcriptional regulation was only responsible for 20%–50% of the observed ﬂux changes. A similar analysis [51] led to the assignment of different *roles* to the regulated enzymes in glycolysis in yeast: regulation of some is predominately hierarchical, for others it is metabolically. For some, the regulation is cooperative between both, and for others it is antagonistic. In an earlier study they compared other central metabolism pathways and found strong qualitative correspondence between transcript and ﬂux changes for the maltose metabolism, partial correspondence for triose-phosphate cycle and pentose-phosphate pathway, and little correspondence for glycolysis [52]. Their results put the prediction methods reviewed in the next chapter into perspective. However, glycolysis is a quite special pathway due to the large enzyme concentrations. Its fast response (for instance, to the sudden loss of membrane potential due to a rupture) is absolutely necessary as ATP depletion leads to rapid cell death. The transcriptional regulation is too slow for this life-saving response. Furthermore, the rapid growth of yeast on a glucose-rich media is an extreme condition rarely found *in vivo*, thus, it is likely that the structure of the metabolic system is not optimized to this situation. So their ﬁndings regarding glycolysis do not seem to be sufﬁcient to discard the idea of observing metabolic changes from transcript data for the entirety of the metabolism. 

### 2.7. Regulation of Metabolic Genes

Which metabolic genes are regulated at all? Wessely *et al*. analyzed transcript proﬁles of *E. coli* and found that pathways (*i.e*., the set of biochemical reactions necessary to perform a speciﬁc metabolic conversion) associated with high protein cost are “controlled by ﬁne-tuned transcriptional programs” and those with low protein cost are only regulated in key reactions [53]. 

And how are the genes (resp. transcription factors) controlled? In the transcription factor network of *E. coli*, a hierarchy of general and speciﬁc transcription factors has been found, and each metabolic function is controlled by a distinct combination of them. Enzymes catalyzing sequential reactions are co-regulated by the same transcription factors, while the regulation at junctions in the metabolic network is more complex [54]. An interesting fact has been found by Notebaart *et al*. which provides an argument to analyze a metabolic network with respect to metabolic functions and not the graph structure alone: “The co-regulation of metabolic genes is better explained by ﬂux coupling than by network distance” in *E. coli* [55]. 

### 2.8. Genetic Interactions

In the studies reviewed so far, the focus was the correlation between an individual RNA and the protein, ﬂux, or concentrations. There is also another form of interactions called epistasis, which has also been modeled in the context of metabolic networks. An epistatic interaction occurs if the phenotypic impact of the knockout of one gene depends on the knockout of another gene [56]. Such an interaction might be caused by redundant reaction paths in the metabolic network in which case it can be predicted by network-based approaches [57,58,59,60]. One common ﬁnding is that most epistatic interactions are restricted to certain conditions [57,59]. Potentially, the veriﬁed set of epistatic interactions can be used for the more accurate interpretation of transcript proﬁles. Szappanos *et al*. studied genetic interactions for the metabolic genes using the ﬂux-balance framework for yeast [61]. They found many “instances of genetic interactions ... not explained by the structure of the metabolic network”, indicating that this is one more complicating factor that has to be taken into account for the mechanistic description of the transcriptional regulation of the metabolism. 

## 3. Systematic Comparison of Methods

To systematically assess the multitude of studies relating RNA proﬁles to the metabolism, criteria will be given to distinguish how the proﬁles are used. 

### 3.1. Absolute/Relative/Coexpression

Expression proﬁles can either be used in several ways. (i) Expression proﬁles can be directly used to assess a single state, which is called *absolute*, e.g., to decide whether a gene is active [62]. (ii) Differential expression proﬁles can be used to differentiate between states (changed conditions, time series)—normally logarithmic expression values are subtracted, which is called *relative*, e.g., to quantify changed metabolic activities [63]. (iii) A third alternative is to analyze the correlation of expression changes for each pair of genes, called *co-expression*, e.g., to assert which metabolic paths are controlled concertedly [64]. 

Absolute expression proﬁles are widely used to predict the active regions in metabolic networks [62,65,66,67,68,69]. Absolute expression proﬁles are also used for network reconstruction [7,8,70]: if a particular gene is expressed in at least one of a large number of expression proﬁles in a particular cell type, then the reaction catalyzed by or the transport process facilitated by its gene product can be considered as a part of the network [71]. 

Relative expression proﬁles are often simply analyzed by counting the number of up-or down-regulated genes using a threshold on the ratio (e.g., more than 2-fold change) or the signiﬁcance level (e.g., using t-test) with respect to classiﬁcations such as gene ontology [72] or KEGG maps [73]. However, a quantitative prediction of the change of the metabolic mode of operation has also been demonstrated [63,74]. To cope with the non-linear relationship of transcript change and enzyme activity change, a ranking approach called Differential Rank Conservation (DIRAC) has been successfully applied [75]. 

Expression correlations are used to determine which genes are commonly regulated, for instance to predict transcription factors. Metabolic pathways with a high correlation of genes coding the necessary enzymes can be considered as a functional mode of operation in a particular cell type [64,76,77]. Ihmels *et al*. analyzed the co-expression of genes coding enzymes and found higher correlations along linear reaction paths between branch points and a hierarchical modularity of the regulation [78]. Loraine demonstrates the use of the gene clustering tool CressExpress for metabolic genes [79]. 

### 3.2. Thresholds

The distinction between active and inactive genes is crucial for all methods using *absolute* expression proﬁles. 

Hebenstreit *et al*. gave clear evidence that in reality there is a clear distinction between genes which are expressed and those which are not expressed (in the sense that the gene product is present in sufﬁcient abundance to take effect in the cell) [80]. The observable concentrations of RNA species is distributed in a bimodal distribution reﬂecting a normal distribution for both expressed and non-expressed genes. To decide whether a gene is considered active or not, a *threshold* is the method of choice. As there is an overlap of RNA abundance levels of *inactive* and *active* genes [80], methods applying the threshold must be robust enough to cope with a certain fraction of wrongly assigned activities. This robustness will also allow the use of transcript data which are not accurately representing RNA counts. Based on a comprehensive analysis of a gene chip in conjunction with proteomics data, an optimal threshold can be calculated. However, mostly such experiments are considered too elaborate and the threshold is set heuristically. Instead, the approach is validated by the overall predictivity. 

The negative effect of uncertainty of the optimal threshold value is reduced by its “soft” application. For instance, in the GIMME algorithm [62] the threshold is applied in such a way that an expression below the threshold entails a gradual (linear) penalty for an activity of the assigned reaction. Thus, a reaction assigned to a gene expressed at a lower level than the threshold can still be considered active but the total amount of these errors is minimized. In the iMAT approach [67,68,81] the threshold application is softened by the introduction of two threshold values. The upper threshold separates the genes highly likely to be active while the lower threshold separates the genes highly likely to be inactive, leaving a range of expression values without a clear attribution. As there is still no guarantee to avoid incorrect gene assignment, an optimization is used where the *clearly active* genes receive a bonus, the *clearly inactive* genes a penalty. More sophisticated is the MADE approach [74] that avoids the arbitrariness of the heuristic threshold setting. For each gene, a single but ﬂexible threshold is calculated from a set of expression proﬁles by identifying the largest gap of values. 

In other approaches, the setting of a threshold is completely circumvented and the expression values are used in a continuous way [69,82,83]. 

### 3.3. Representation of the Metabolic System

The way the metabolic system is represented is another important aspect of the methods. Mostly, the system is represented by the metabolic network which consists of the metabolites and the biochemical reactions which convert the metabolites in ﬁxed quantities, the stoichiometric factors—thus, it is called stoichiometric model. Often a stoichiometric model is used to compute ﬂux distributions in the ﬂux-balance framework [62,65,69,74]. A different approach is to use metabolic paths (small linear chains of reactions) which do not necessarily form a complete network [64,84]. An alternative way to represent the metabolic system is to compute the set of elementary ﬂux modes ﬁrst [40] and perform the analysis using these ﬂux distributions [41]. Also the decomposition of the total ﬂux as convex sum minimal ﬂux modes [85] parameterized by gene expression has been proposed [63]. The ﬂux balance framework is equivalent to a bipartite graph (Petri net [86]) but also simpliﬁed graphs have been used such as the adjacency graph [87]. 

In a simpliﬁcation of the stoichiometric model, the stoichiometric factors are ignored [88]. Hancock *et al*. use such a graph representation where the nodes are the metabolites, and for every biochemical reaction an edge is drawn from each substrate to each product [38]. 

### 3.4. Type of Inference

A ﬂux distribution or an active subnetwork can be computed by penalizing ﬂuxes belonging to *inactive* genes and/or bonusing nonzero ﬂuxes belonging to *active* genes (in other words, the binary compliance to the expression proﬁle) [62,67,81,89]. This approach has also been used as a secondary criterion in constrained ﬂux-balance optimization [68]. Expression data has been used to deﬁne upper bounds on ﬂuxes in a ﬂux-balance computation [69]. Another possibility is to use the expression values to deﬁne target values for the ﬂuxes and minimizing the quadratic deviation [82]. A similar method, based on error minimization and developed for protein levels [90], can in principle also be applied to expression data. A multi-layer probabilistic framework, called PROM, mainly integrates a metabolic network with a transcriptional regulatory network but is also capable of using transcript data [83]. Its basic idea is to assign a probability value to gene states. Expression proﬁles have been used to *rank* reaction paths [64] or similarly “metabolic modules” [91]. Based on the textbook pathway deﬁnition (e.g., implemented in KEGG [73]), expression values have been used to score pathways in a framework called differential rank conservation [75,92,93]. The clustering of sets of genes, very common in the elucidation of transcription factors, has also been applied in conjunction with metabolic functions [87]. Also, graph theoretical inference has been used [94]. The topology of the metabolic network is the starting point to ﬁnd the so-called regulatory signatures, patterns of gene changes indicating a diseased state (type 2 diabetes mellitus in this case) [95]. 

Gene set analysis [96] can be applied to metabolic pathways [97] as a distinct approach to use transcript correlation. A common technique to evaluate transcript proﬁles is to count up-/down-regulated genes (with a signiﬁcance threshold); this can also be applied to KEGG pathway maps [73] or GO terms [72] to estimate the emphasis on certain functional characterizations [98]. 

### 3.5. Biological Focus of Studies

Methods applying transcript data to the metabolism can have many different aims. As a distinguishing characteristic, some studies are directed to lay theoretical foundations, while others are directly targeted to answer speciﬁc biological question. 

For the ﬁrst category, the reconstruction of a metabolic network for a speciﬁc cell with the help of transcript data can be mentioned [71]. Once a universal metabolic network is reconstructed (such as the universal human cell [7]), the subnetwork of reactions in speciﬁc cell type can be obtained with the same approach [8,67,81,89]. Similarly, transcript data is also used to estimate the set of active reactions in a particular state [68,69,82,99]. From the set of active reactions in a particular state, the essential information can be extracted in a further processing step, such as the so-called ﬂux phenotypes [48] or, similarly, the metabolic state [90]. The detection of novel metabolic pathways [100,101] is an application in the area of fundamental biochemistry. 

There are a number of studies that try to understand the regulation patterns by analyzing the co-expression of metabolic genes in a large number of transcript proﬁles [38,39,54,55,78]. These regulation patterns can lead to the prediction of transcription factors of one or several genes. Reed and Palsson analyzed the connection between correlated genes and coupled reactions [102]. 

Some applications of transcript data are directly related to clinical questions such as the prediction of biomarkers [17,94], the prediction of drug targets [67,68,103], identiﬁcation of reporter metabolites in type 2 diabetes [95], the study of the effects of a drug such as baicalein [104], and identifying diet effects [105]. The search for target metabolites of regulation (*i.e*., concerted regulation of genes to change the concentration of a certain metabolite) was the focus of another study [38]. If the underlying hypothesis of this study was true also for organisms other than *E. coli*, then this method would open a path to identify biomarkers in biotechnology and medicine. 

Often, studies have an explicit biotechnological focus, for instance plant strain optimization [106], bacterial production rate optimization [107,108], optimization for algal growth [109,110], and understanding seed ﬁlling of oilseeds [42]. 

## 4. Available Software

The threshold-based activity prediction GIMME [62] (and the closely related iMAT [81], see Section 3.2 for the difference) is widely used, as it requires only minimal preconditions: a functional stoichiometric model and a few transcript proﬁles sufﬁce. Without any further requirement it can be applied to predict the exometabolic ﬂuxes. As these ﬂuxes are often known, they can be used to increase the reliability of the model, in a multi-step algorithm to ensure the concordance to the input/output ﬂuxes [62,81] or directly in the ﬂux-balance optimization [68]. 

These expression-based prediction methods have been implemented in the universal ﬂux computation frameworks COBRA [111] and FASIMU [112]. For the iMAT method [81] a standalone implementation is available [113]. The software for quantitative application of transcript data for ﬂux prediction by Lee *et al*. is also freely available [82]. The TIGER [114] toolbox can be recommended if transcriptional regulation should also be taken into account. If a large number of transcript proﬁles are available and transcriptional networks should also be modeled, the freely available probabilistic framework PROM [83] can be recommended. The threshold value can be adjusted if quite a number of transcript proﬁles are available. It can be calculated individually with an optimization using MADE, which is also freely available [74]. 

To analyze correlations of the expression of different genes from transcript proﬁles with respect to metabolic paths, the PathRanker method [64] offers a freely available implementation. It does not require a functional stoichiometric model but needs large proﬁle sets to work reliably. 

## 5. Conclusions and Outlook

Inferring metabolic activity changes from transcript proﬁles is justiﬁed in two ways: mechanistically and by the assumption of evolutionary optimality. The former is based on the fact that RNA is translated into proteins then working as enzymes or transporters, thus modifying the metabolic ﬂux related to the function. The latter is based on the argument: if the cell undertakes the effort to increase the mRNA production rate, it does so only with a purpose (related to the philosophical concept of *ﬁnal cause*). The most likely purpose is to enhance a function for which the coded protein is required. 

As the direct correlation of transcript proﬁles to metabolic reaction ﬂuxes is not high, there is a wide range of applied methods having different strengths and weaknesses. The critical question is whether a particular method is suited to a particular application. 

There is a clearly recognizable trend that the applied methods are increasingly enriched with available knowledge as the only way to increase the predictivity. 

For the outlook of the ﬁeld, it is foreseeable that large-scale metabolomics, proteomics, ﬂuxomics, and enzyme characterization will become more manageable and affordable and the need to cover the wide distance from transcript to metabolism will vanish. The methods can then be improved with mechanistic descriptions of the underlying processes as soon as they are discovered. The methods crossing several layers will have to include more components as it will be possible to parametrize them using experimental data. Genome-scale quantitative proteomics is on the brink of being widely available and feasible [11]. Quantitative metabolomics has reached the level of feasibility for hundreds of species. The developers of the reviewed methods and the users of their results will adopt this data when the coverage, cost, or accuracy makes it viable to do so. The application of mRNA data is, at the current time, just the most applicable means. 
